# Neural plasticity in early potters: Shape analysis and TMS-EEG co-registration trace the rise of a new motor skill

**DOI:** 10.1371/journal.pone.0316545

**Published:** 2025-01-17

**Authors:** Vanessa Forte, Luisa Sartori, Antonino Visalli, Mustafa Yildirim, Gaspare Galati, Massimo Vidale, Emanuela Faresin, Antonino Vallesi

**Affiliations:** 1 Department of Science of Antiquities, Sapienza University of Rome, Rome, Italy; 2 Department of Cultural Heritage: Archaeology, History of Art, Cinema and Music, University of Padova, Padua, Italy; 3 Department of General Psychology, University of Padova, Padua, Italy; 4 IRCCS San Camillo Hospital, Venice, Italy; 5 Department of Cognitive Psychology, Ruhr University Bochum, Bochum, Germany; 6 Department of Psychology, Sapienza University of Rome, Rome, Italy; 7 Department of Neuroscience, University of Padova, Padua, Italy; 8 Padova Neuroscience Center, University of Padova, Padua, Italy; Israel Antiquities Authority, ISRAEL

## Abstract

In this study, we explored the biocultural mechanisms underlying ancient craft behaviours. Archaeological methods were integrated with neuroscience techniques to explore the impact on neuroplasticity resulting from the introduction of early pottery techniques. The advent of ceramic marked a profound change in the economy and socio-cultural dynamics of past societies. It may have also played a central role in developing new craft skills that influenced the neural plasticity of the potters. Coiling, one of the most widespread neolithic techniques, requires precise hand movements and the ability to regulate finger pressure to shape the clay without deformation. In a pilot study involving intensive training in neolithic pottery, we used TMS-EEG co-registration to monitor a group of participants and we examined the shape of the artefacts they made before and after training. Our findings suggest changes in the functional properties of the primary motor cortex (M1) responsible for the control and execution of actions. We also observed an improvement in symmetry and consistency of the artefacts and a significant reduction in errors. This multidisciplinary approach sheds light on the mechanisms of material culture’s variation in the archaeological field and provides promising insights into the co-evolution of technology and human skill.

## Introduction

Pottery is widely recognized as a neolithic technological innovation. Nevertheless, the interaction of humans with clay-based materials is much older. Early evidence found in late Pleistocene and early Holocene contexts consists of structures, animal-shape reproductions, human figurines [1,2] made in clay and, in some hunter-gatherer groups, even ceramic containers [3–5]. This evidence, scattered over time and space, represents the output of an early experience of humans in exploring the potentials of a plastic raw material which keeps its shape after drying and changes its physical features when exposed to high temperatures, converting into ceramic [6]. The innovation of ceramic technology impacted not only the economy of prehistoric communities but also their social and cultural spheres. It influenced various aspects such as food production and consumption, commensality and, most likely, the emergence of new skills and craft gestures.

According to archaeological evidence, the earliest pottery productions were handmade without rotating kinetic energy (*sensu* Roux) [7] and using modelling techniques that likely involved pinching and lifting from single clay lumps, the assemblage of flat slabs, or the rolling of elongated lumps of clay to shape coiled pots [7–9]. The present research focuses on this latter technique, known as coiling, because, as for general agreement, it is the most widely practised approach in early pottery productions across economic, social and cultural contexts [5,7,10–12]. The entire creative process of coiling required the use of fingers and a toolkit consisting of bone, wooden spatulas or pebbles [13,14] to overlap, pinch coils and homogenise irregularities along the clay surfaces [7,14,15]. Shaping pottery is often mistakenly regarded as a straightforward skill that can be easily acquired by anyone, especially when compared to flintknapping or, remaining in the realm of clay-based materials, to wheel-throwing. Hand-working with clay, instead, is likely to require a skill that is developed over time, often starting from childhood, when children and adolescents embody motor skills, technological concepts and procedural knowledge, through scaffolded processes of training, by observing and imitating adults or actively participating in the steps of the production process for creating small figurines or miniature objects out of clay [7,16–18] and decorating their surfaces [19].

The characteristics of the archaeological ceramics suggest that dexterity and refined technical knowledge were not skills developed equally well by all the individuals in a community [15,20]. The debate on diverse aspects of the emergence of expert behavior, such as onset and mechanisms, is still open. As observed in previous studies, advancement in a practical domain can be influenced by several conditions such as previous experience and inherited know-how, mobility, social context and self-motivation [21,22].

The combination of motor skill and degree of technical knowledge, defined as the correctness in performing a sequence required by a given technique, influenced the variability of the material culture produced by potters [23]. Tracing this variation has therefore been an overarching aim of archaeologists interested in craft skill [24]. Nevertheless, studies connecting the neural activity underpinning skilled actions and the evidence of skilled productions in archaeological contexts are rare [22,24–26] and they represent a novelty for the ceramic study tradition. Behavioral studies on potters have been carried out so far in traditional contexts, with a specific focus on wheel-throwing productions [7,27–29]. These works revealed complex mechanisms involved in acquiring motor skills related to wheel throwing. According to these and other studies, what differentiates people who develop and embody a new set of skills is the capacity to manage and overcome the constraints imposed by the raw material and the manufacturing sequence, and this improvement is measurable in a gradual reduction of errors along with a growing dexterity of gestures [7,14,30–32].

Cognitive neuroscience studies suggest that the embodiment of a set of skills can affect neuroplasticity [33,34] and, in the case of craft activities, potentially modulate structural and aesthetical features of the material production. Hence, the gradual transition from working with stone (through reductive sequences) to working with clay (mostly with additive sequences) may have fostered the development of new skills. These emerging skills, in turn, may have changed the functional (e.g., electrophysiological) properties of brain areas responsible for action control and execution, including the end-point of the cortical motor system, namely the primary motor cortex. For the first time, in this contribution, we aim to trace the impact on neuroplasticity resulting from the emergence of early ceramic production techniques. To this end, in this pilot study, we have applied a novel neuro-archaeological approach that combines experimental archaeology, cognitive neuroscience and behavioural techniques. A group of participants naïve (i.e., unfamiliar) in shaping coiled vessels was trained in pottery using raw materials, techniques, and knowledge transfer conditions as close as possible to those putatively attributed to early pottery production aiming at developing coiling motor skills.

We analysed the changes in shape variation of the handicrafts produced by the participants before and after the training and the participants’ neural indices through the simultaneous recording of motor-evoked potentials (MEPs) from the muscle involved in the abduction of the index finger (i.e., first dorsal interosseus) and the muscle involved in the thumb opposition (i.e., opponens pollicis) of the right hand—induced by single-pulse transcranial magnetic stimulation (TMS) on the primary motor cortex, and electroencephalogram (EEG), a simultaneous combination of techniques known as TMS-EEG co-registration [35,36]. TMS-EEG co-registration has a high potential as the EEG allows us to have a more global image of the brain activity that is generated by the magnetic field of the TMS and to explore neural correlates of observing actions of pottery production before and after training.

We exploited a classical TMS paradigm based on the observation of correct and incorrect actions by experts or non-experts in which an effect of motor expertise on corticospinal excitability was demonstrated at the onset of the incorrect action (i.e., motor facilitation in the group with motor experience [37]). In this case, we adopted videos that showed participants clips of correct and incorrect actions related to the production of clay coils. We analyzed changes in the shape and qualitative characteristics of the objects modelled by the participants at the beginning and the end of the training. Then, we integrated these observations into the neurophysiological (EEG and MEP) signals to assess whether this specific motor skill and the quantitative and qualitative variations we observed in the material production were associated with neuroplasticity. In terms of motor expertise, we predicted an increase in the symmetry and consistency of the coils when comparing production before and after training, and across groups (trained, non-trained). In neural terms, we hypothesized a change in the motor cortical representation of trained hand muscles after intensive training of pottery, so that observation of videos with a technical error should reflect in the modulation of corticospinal excitability.

In order to discriminate between a muscle-specific error or a generic error of the tool, we adopted an experimental condition in which participants observed the (correct or incorrect) working of a clay coil with the hands, and a control condition in which they observed a (correct or incorrect) technique of smoothing the clay with a spatula, such that the error did not imply a specific movement of any muscle. In particular, the video of the first technique showed a specific muscular error (i.e., excessive pressure) exerted by the index finger (but not the thumb), while the video of the second technique showed an error in the use of the tool which did not imply any differential activation of the two muscles. We implemented several levels of experimental control: a control group (no training), a control condition (spatula), a control video (correct performance), and a control muscle (OP) normalised to the control baseline. We, therefore, expected that our experimental paradigm would be able to detect extremely specific effects if they existed. Concerning the EEG, we expected to observe between-group differences in the EEG correlates of action and error observation after the training, as well as in the TMS-induced brain excitability.

## Materials and methods

### Participants

We recruited twenty-eight young healthy volunteers in the bachelor’s and master’s degree programs in Archaeology at the University of Padova, Italy. The sample size for this pilot study was determined considering time constraints imposed by the project timeline, rather than a formal power calculation. A sensitivity analysis showed that it was possible to observe a between-group difference corresponding to an effect size d = 1.12 (alpha = .05, power = .80). Participants’ handedness was assessed using the Edinburgh Handedness Inventory (EHI, Oldfield, 1971). They were all screened for the TMS exclusion criteria and for neurological, psychiatric and medical problems [38–40]. The experimental group consisted of 14 participants (one of them was then excluded due to technical problems: 8 females and 5 males, 12 right-handed, average age: 22.08, SD = 3.5) with no experience in pottery (**S1 Table**). They attended a neolithic pottery technique training (coiling). During this period, they used raw materials and tools mimicking the neolithic ones to create a neolithic-style pot with specific size parameters. The control group—with no practical experience in pottery modelling, consisted of 16 participants (6 females, 10 males, 13 right-handed, 1 left-handed and 1 ambidextrous, average age: 21.25, SD = 1.3). All participants gave their informed written consent to participate in the study, which was conducted following the ethical standards of the 2013 Declaration of Helsinki for Human Studies of the World Medical Association. The recruitment started the 1^st^ of September 2022 and concluded the 19^th^ of October 2022. The project was approved by the Ethical Committee for the Psychological Research of the University of Padova (approved protocol reference number: 4917).

### TMS-EEG co-registration

Two sessions of TMS-EEG co-registration were carried out at the beginning and at the end of the training, involving both the training and the control groups. The time interval between the two sessions was 44 days on average (range of days = 33–63, SD = 16.8).

The EEG data were recorded (sampling rate = 5000 Hz; online filter = 0.1–1000 Hz) using TMS-compatible BrainAmp amplifiers (Brain Products, Germany) from 64 Ag/AgCl electrodes mounted on a TMS-compatible elastic cap (EASYCAP GmbH, Germany), using the 10/10 system. Impedance was ≤ 10 kΩ. All electrodes were referenced to FCz during the recording, while AFz was used as ground.

### Stimuli

**Fig 1:**
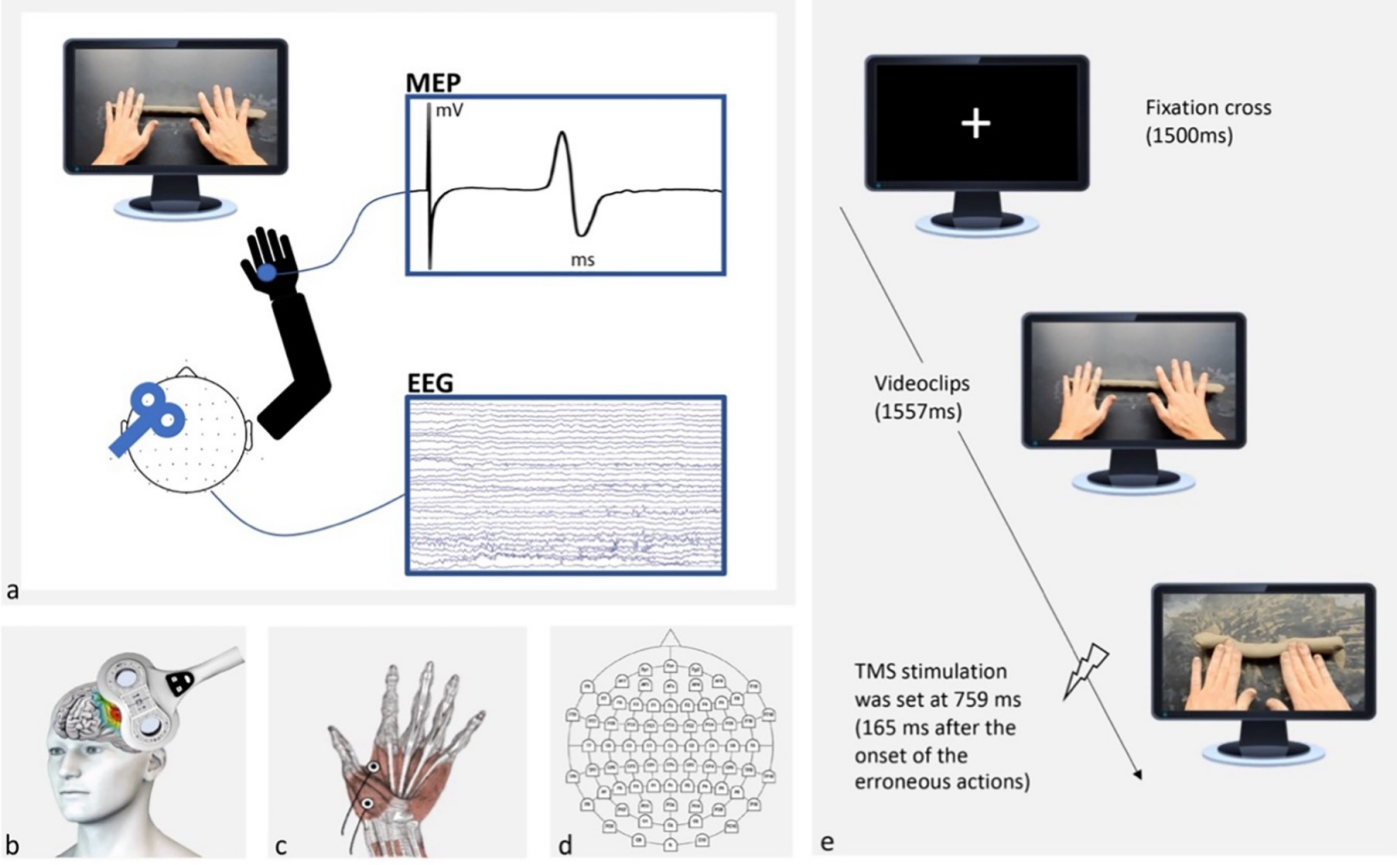
(a) Experimental setup. Participants were shown videoclips of correct and incorrect actions related to the production of clay coils, while a TMS-EEG co-registration was performed. A TMS-induced MEP from a representative participant is shown in the upper box, while an EEG recording is shown in the lower box. (b) A TMS figure-of-eight coil was placed over the participant’s left M1 to investigate corticospinal excitability during action observation. (c) TMS‐induced MEPs from the FDI and OP hand muscles were recorded through pairs of surface electrodes. (d) A TMS-compatible elastic cap with 64 Ag/AgCl electrodes was used to record cortical electrical activity. (e) Timeline. A fixation cross appeared for 1500 ms, followed by videoclips presentation for 1557 ms. Timing of single-pulse TMS stimulation was set at 759 ms for all conditions (i.e., 165 ms after the onset of the erroneous actions).

Four video-clips were used as experimental stimuli (**Fig 1**). The videos depicted a right-handed female pottery expert performing two techniques:

1. Correct Coil Making. The ceramic expert performed a correct rolling motion of her left and right hands on the clay cylinder.
2. Incorrect Coil Making. The ceramic expert performed a correct rolling motion of her left and right hands on the clay cylinder, but at the end of the video she made a clear mistake: she pressed her index finger too hard on the cylinder and produced a deformation of the coil. The error occurred 594 ms after video onset.
3. Correct Surface Treatment. The ceramic expert reached out and grasped a spatula placed on the table with her right hand and brought it close to a clay vessel held in her left hand to smooth the surface.
4. Incorrect Surface Treatment. The ceramic expert reached out and grasped a spatula placed on the table with her right hand and brought it close to a clay vessel held in her left hand to smooth the surface but, at the end of the video, she pressed the spatula too hard on the vessel and produced a scratch. The error occurred 594 ms after video onset.

The video-clips were filmed from a 1^st^ person point of view with the use of a Canon Legria HFM36 (Tokyo, Japan). Videos were modified in post-production to show the very same frames until the onset of the incorrect gesture. Each stimulus presentation lasted 1557 ms and the animation effect was obtained by presenting each frame 33 ms in series. The last frame lasted 600 ms. The dimension of each stimulus was 1,024 x 768 pixels displayed on a 24-inch monitor (resolution: 1,440 x 1,080 pixels, refresh rate: 120 Hz, color depth: 32 bits). Each frame was presented in the centre of the screen with a black background.

### Training

Participants with no experience in pottery making were asked to take part in a pottery training during which they learned to shape homogeneous coils, overlap them to shape the vessel and refine the vessels’ surfaces using bone and wooden spatulas.

Teaching conditions were inspired by knowledge transmission systems likely adopted by prehistoric communities (e.g., practising in groups and imitation) [41]. An expert with 20 years of experience in coiling techniques trained the naïve participants through voice instructions and showed them the technique for shaping coils and modelling and refining vessels. In each class, the expert worked among the participants without interfering with their work and their products. This allowed each participant to follow their own pace. The raw material selected for the experiment was a non-commercial calcareous clay paste with a fine granulometry collected from nature, grounded and mixed with water to ensure a texture and working conditions compatible with early pottery productions. All the participants used the same clay and tools for the whole duration of the training. Each participant was asked to: 1. Shape a set of 10 coils at the beginning and the end of the training mimicking rolling movement with both hands; 2. Shape coiled vessels mimicking a neolithic-style pot (an open shape with approximately 10 cm in diameter at the bottom, 20 cm in height, 15 cm in width, and 1 cm in thickness), 3. Smooth and refine the vessel surfaces using bone spatulas inspired by toolkits used by neolithic artisans. The participants were asked to produce and refine at least one vessel during each class, a total of 100 vessels were shaped. Classes were held twice or three times a week with the constraint to reach at least 24 hours of practice during a four-weeks period.

### Procedure

Before and after the training the participants took part in two TMS-EEG co-registration sessions. The volunteers were asked to sit on a comfortable chair in front of the computer that was used for the presentation of experimental stimuli while the MEP and EEG signals were recorded. During the experimental session, participants were instructed to relax and watch the videos trying to minimise movements of their eyes and head. The study consisted of 4 blocks with 16 trials each, a total of 64 trials, and 16 repetitions of each video clip. At the beginning of each block, a text message was displayed on the screen for 5000 ms, reminding participants to relax and refrain from moving their eyes and body, followed by a central white fixation cross on black background (Courier New, Bold, font 80), which lasted for 250 ms. This procedure ensured all participants started observing the video stimuli from the same foveal center point. Before and after each block, 4 TMS pulses were delivered to compute the intra-session MEP baseline and, during the task, the TMS pulse was delivered once every two trials to comply with the optimal discharge frequency according to the single-pulse stimulation guidelines [39,42] while optimising the EEG sampling. TMS was administered in half of the repetitions of each video clip. Each trial in the experiment started with the presentation of a fixation cross, which lasted for a duration of 1500 ms. Subsequently, video clips were displayed at the centre of the screen. Following a precise timing protocol, TMS pulses were administered 759 ms after the initiation of the video clips (i.e., 165 ms after the onset of the erroneous action). The order of blocks and conditions was randomized for each participant. Participants were asked if they needed a break between blocks to ensure that they could sustain their attention throughout the experiment. The experimental task was designed and run with the use of E-prime software (Psychology Software Tools, version 2.0).

### Electromyography (EMG)

Surface EMG activity was recorded simultaneously from the first dorsal interosseous (FDI) and opponens pollicis (OP) muscles of the participant’s right hand through two pairs of Ag/AgCl electrodes (1 cm diameter) placed in a belly-tendon montage (**Fig 1**). After the skin was cleaned, electrodes with a small amount of water-soluble EEG conductive paste were placed and fixed on the target positions. The active electrode was placed over the belly of the muscle, determined by palpation during maximum voluntary contraction, and the reference electrode was placed over the proximal interphalangeal juncture. The ground electrode was placed on the participant’s right wrist. The electrodes and wires were secured and placed so as not to restrict the participant’s movements and assure comfort. The skin impedance, evaluated at rest prior to beginning the experimental session, was considered of good quality when below the threshold level of 5 kOhm. The electrodes were connected to an isolable portable ExG input box linked to the main EMG amplifier for signal transmission via a twin fiber optic cable (Professional BrainAmp ExG MR, Munich, Germany). A high-pass filter of 30 Hz and a low-pass filter of 1000 Hz were applied to the raw myographic signal, which was amplified prior to being digitalized (5 kHz sampling rate) and stored on a computer for off-line analysis. EMG signals were recorded with Brain Vision Recorder software (Brain Products GmbH, Munich, Germany).

### Transcranial magnetic stimulation (TMS)

Single-pulse TMS was administered using a 70 mm figure-of-eight coil connected to a Magstim BiStim2 stimulator (Magstim Co., Whitland, UK). Pulses were delivered to the left primary motor cortex (M1) of the participant, in correspondence with the representation of the right hand. The TMScoil was placed on the head at a 45-degree angle relative to the interhemispheric fissure, with the handle pointing laterally and caudally [43,44]. The optimal scalp position (OSP), which is defined as the best position for the TMS coil on the scalp at which the lowest intensity of stimulation elicits the largest MEP of both thumb and index fingers muscles, was determined by moving the TMS coil in approximately 0.5 cm steps around the presumed hand motor area. The OSP was then marked on a tight-fitting cap worn by the participants, ensuring a correct coil placement throughout the experiment. During the experiment, the TMS coil was held on a tripod and the experimenter continuously checked the position to maintain a constant positioning with respect to the marked OSP. For each participant, the resting motor threshold was determined as follows: the lowest stimulation intensity able to induce MEPs of at least 50 μV peak-to-peak amplitude in a relaxed muscle in 5 out of 10 trials [45] in the less excitable muscle to record a clear and stable MEP signal from both muscles throughout the experiment. The stimulation intensity was set at 110% of the resting motor threshold to measure MEPs peak-to-peak amplitude (mV). The inter-pulse interval was longer than 6 s, thereby avoiding changes in corticospinal excitability due to repeated TMS pulses [42]. TMS stimulation and EMG recording were managed by E-Prime V2.0 software (Psychology Software Tools Inc., Pittsburgh, PA, USA).

### Data analysis

#### MEP data

Individual peak-to-peak MEP amplitudes (mV) were analysed offline using Brain Vision Analyzer (Brain Products, BmbH, Munich, Germany). The peak-to-peak MEP amplitude for the FDI and OP muscles was calculated as a measure of the corticospinal excitability of each participant. Trials in which EMG activity greater than 100 μV was present in the 100 ms window preceding the TMS pulse were discarded to prevent contamination of the MEP measurements by background EMG activity (<1%); in addition, values greater than 3 SD of the mean were excluded as outliers (<5%). To control for inter-individual variability in MEP amplitudes, separately for each participant and each muscle, the raw MEP amplitudes were normalized computing a ratio between MEP amplitude values recorded during the experimental conditions and during the intra-session baseline blocks. To mitigate the influence of unbalanced number of observations per conditions, statistical analyses of MEP for FDI and OP muscles were conducted by means of random-intercept only linear mixed-effects models (LMM) using the *lme4* package [46] in R (http://www.R-project.org/). Specifically, for each muscle, we specified the following model: MEP ~ GROUP x SESSION x TECHNIQUE x CORRECTNESS + (1|Participant). The fixed part thus included main effects for Group (Trained vs. Control) Session (Pre vs. Post), Technique (Coil Making vs. Surface Treatment), Correctness (Correct vs. Incorrect) and all their interactions. Since this was a conceivably underpowered pilot study with an exploratory aim, we also conducted uncorrected post-hoc pairwise comparisons using the *grafify* R-package [47].

#### EEG pre-processing

All pre-processing and statistical analysis for EEG data were performed by using MATLAB (The Mathworks, Inc.) software using scripts from EEGLAB [48] toolbox. The analysis of concurrent EEG and TMS followed the one described in Rogasch and colleagues [49]. We did not perform temporal alignment to TMS pulses because for half of our trials there was a TMS pulse and for the other half there was no TMS pulse. To enhance the performance of Independent Component Analysis (ICA), the TMS pulse artifact and the peak of TMS-evoked muscle activity within the time range of 0 (TMS trigger) to 6 ms were replaced with constant amplitude. Upon visual examination, it was determined that there were no disconnected electrodes or trials exhibiting significant, non-repetitive artifacts. Consequently, no manual rejection of channels or epochs was conducted during this stage, as it was deemed unnecessary based on the visual inspection results. At this stage, it was observed that residual TMS-evoked muscle and electrical/movement artifacts persisted in the data, extending beyond the initial 6 ms time frame that was previously removed. To address this issue, a preliminary Independent Component Analysis (ICA) was conducted using the FastICA [50] algorithm, following the recommendation of Rogasch and colleagues [49]. This ICA aimed to identify and manually select independent components (ICs) that specifically corresponded to these lingering TMS-induced artifacts, allowing for their effective removal from the data.

The presence of these artifacts can have a negative impact on the accuracy of ICA when used for purposes such as recovering neural activity or eliminating physiological artifacts. Additionally, by removing these artifacts, it becomes possible to apply filtering techniques, potentially further enhancing the effectiveness of ICA decomposition [51]. On average, 1.6 ICs were removed at this stage (SD = 0.9, range = 0–5). This removal ensured that the EEG data for all participants were no longer contaminated by TMS-evoked muscular artifacts.

To further process the data, several steps were taken. Firstly, the constant amplitude data surrounding the TMS pulse were interpolated using linear interpolation. Next, the EEG data underwent band-pass filtering using a Hamming windowed sinc Finite Impulse Response (FIR) filter, with cut-off frequencies set at 1 and 40 Hz. Subsequently, the FastICA algorithm was applied for the decomposition of ICs. An automated selection of brain components was conducted with the aim of removing residual TMS artifacts and artifacts associated with eye movements, blinks and muscular activity, channel’s noise and other non-brain noise considering the classification results of the ICLabel plugin in the EEGLAB software [52], and the residual variances of the best-fitting equivalent IC dipoles calculated using the DIPFIT plugin in EEGLAB. Specifically, ICs with a brain label probability below 0.7 or a best-fitting equivalent dipole with a residual variance equal to or greater than 0.15 were excluded from further analysis. Prior to the rejection of components, a thorough visual inspection and labelling process were conducted for all the components. On average, 47.2 ICs (SD = 4.5, range = 35–56) were removed during this stage. Data was epoched from -1000 to 2000 ms around the onset of the video clips. The TBT EEGLAB plugin was used for the automatic rejection and interpolation of channels on an epoch-by-epoch basis. Epochs with more than 6 bad channels were excluded, otherwise the (≤6) bad channels were interpolated in that epoch. Channels that were identified as bad in over 30% of the epochs were excluded and interpolated as well.

#### EEG inferential analyses

**Fig 2:**
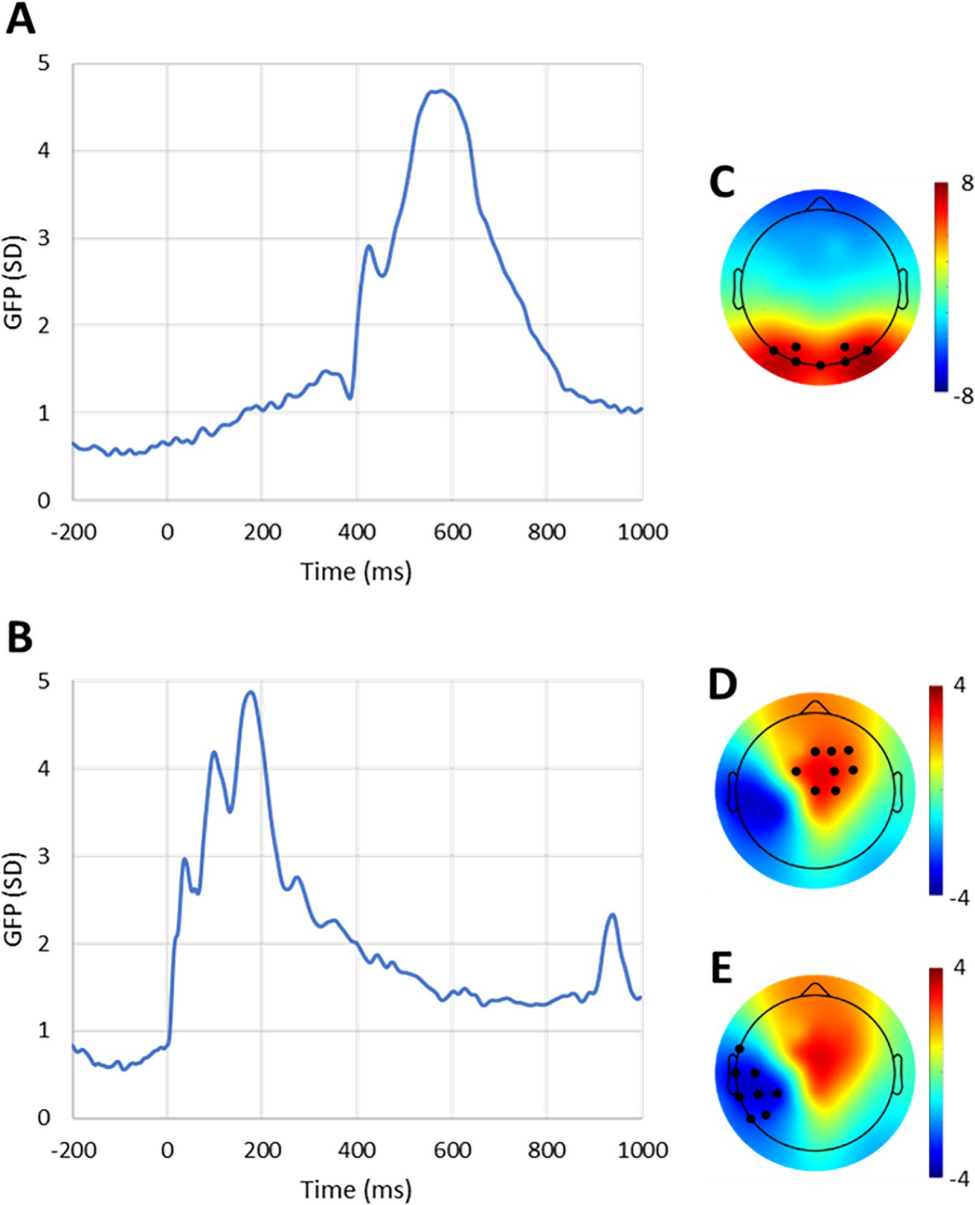
Global field power (GFP). The figure shows the GFP time-locked to the onset of the video clip (A) and to the delivery of the TMS pulse (B). The topoplots show the EEG signal averaged in the time-windows of interest (GFP > 3 SD) across participants. Specifically, Topoplot C shows the grand average EEG in the 478 ms to 682 ms time-window locked to clip onset. The cluster of channels used for inferential analysis (PO7, PO3, PO4, PO8, O1, Oz, O2) is indicated by black dots. Topoplots D and E show the grand average EEG in the 74 ms to 230 ms time-window following the TMS pulse. The two clusters of channels used for inferential analysis (Fz, F2, F4, FC1, FC2, FC4, Cz, C2 in D, and FT7, T7, C5, TP7, CP5, CP3, P7, P5 in E) are indicated by black dots.

Due to the pilot nature of this study, we preliminary computed the Global Field Power (GFP) to identify time windows and channels of interest for our analyses (**Fig 2**). Specifically, GFP is the spatial standard deviation of EEG voltage, and quantifies the level of activity at each time point [53]. GFP was computed for each participant (irrespectively of the experimental conditions) and then averaged across participants. The time windows when mean GFP reached its maximum values were used to find the latencies of evoked potential components [54] to be analysed.

The first observed increase in GFP (>3 SD) occurred within the time interval ranging from 478 ms to 682 ms. This aligns with participants viewing the action before its completion, and during this time-window no errors had yet occurred in trials featuring erroneous actions. Any differential activation detected during this time-window may reflect variations in the mechanisms specifically associated with action observation. After the identification of this time-window of interest, we plotted the topoplot of the EEG signal averaged in this time-window and across participants, to find the channels that were responsible for that increased GFP. The following channels were identified for subsequent analysis in the first time-window: PO7, PO3, PO4, PO8, O1, Oz, O2. For each session, participant and experimental condition, we extracted the EEG signal (baseline corrected to the mean of the 200-ms interval before the video clip onset) averaged across the identified channels and time-points of interest. Then, a 2 groups x 2 sessions mixed ANOVA was conducted (to note, muscle-specific hypotheses do not apply to EEG analysis. As for MEP analysis, we also conducted uncorrected post-hoc pairwise comparisons.

The second window of interest (GFP > 3 SD) corresponded to the time range from 74 ms to 230 ms following the delivery of TMS pulse. Since there was no evident activity in the GFP for non-TMS trials, the analysis in this time window was limited to the TMS trials. Differential activation observed within this second time-window may indicate the involvement of error monitoring mechanisms. Two clusters of channels were identified for subsequent analysis in the second time-window. The first cluster included the following channels: Fz, F2, F4, FC1, FC2, FC4, Cz, C2. The second cluster included: FT7, T7, C5, TP7, CP5, CP3, P7, P5. A 2 groups x 2 sessions x 2 correctness (correct or incorrect) mixed ANOVA was conducted for each cluster. We also performed exploratory uncorrected post-hoc pairwise comparisons.

#### Analysis of the shape variation of coils

*Qualitative analysis*. The qualitative analysis of the coils aims at identifying and localising variations in terms of thinning and thickening of the general shape as a consequence of uncontrolled and repeated hand palm and finger pressure during the rolling-up movement.

*Quantitative analysis*. As an integration to the qualitative analysis of the morphological difference identified on the coils produced before and after the training sessions, we applied a geometric morphometric approach aimed at quantitatively assessing difference in the shape of the coils produced during the two phases. Digital images of 180 coils were processed in tpsDig2 (https://life.bio.sunysb.edu) using the *outline object tool*. This function allows to create outlines made of equidistantly placed semi-landmarks, which are then stored in a.tps file which is then imported in the R environment. The outline data was processed using the Momocs package [55] in Rstudio (v. 2023.12.0+369). Other utilised packages include tidyverse [56] and dplyr [57] for data manipulation, ggsci [58] and ggpubr [59] for data visualisations and readr [57] for importing and exporting the data.

Outline data has been processed through Elliptic Fourier Analysis (EFA). The outlines of the coils were normalised by defining a common centroid and then scaled accordingly, while eventually unclosed shapes were closed if present in the dataset. Following normalisation, the shape of the coils was reconstructed through EFA by computing the harmonic power. For our study we opted for the number of harmonics allowing for the reconstruction of 99.9% of the shape approximation, which were used to generate the outline coefficients.

The Elliptic Fourier coefficients were utilised to define the main differences in the shape of the coils through Principal Component Analysis (PCA). Finally, multivariate analysis of variance (MANOVA) was employed to test the existence of significant difference (alpha level 0.05) between the shape of coils produced before and after the training sessions.

#### Morphometry of the vessels

A representative sample of the objects produced by the participants during the neolithic pottery training was documented through 3D modelling and analysed using a combination of morphometrical quantification and qualitative description of the manufacturing traces. This integrated approach allowed for precise quantification of potential variations in measures, size and accuracy of the objects. 3D modelling was applied through a 3D close-range photogrammetry technique. A standard and consolidated photogrammetric pipeline was adopted. Vessels were placed on a table prepared with the application of targets. At least 60 pictures of every vase were taken, changing the height of the shooting position with locked camera settings and turning objects upside down. These parameters grant overlapping for every shot, taken at a reasonable distance from objects illuminated by diffuse lights, and placed outside of a photographic illumination box. Vessels were acquired using a Nikon Cooplpix S9700 30x camera. The photogrammetric 3D reconstruction, in terms of bundle adjustment, camera orientation, sparse cloud and dense one generation, was carried out using the Agisoft Metashape software. Digital models were first studied and analysed under different synthetic sources of lighting to have a first idea of the surface trend and then they were sliced to get views passing through the planes. This process leads to the production of a faithful representation of the ceramic thickness necessary for the following measurement phase.

Data were processed using R to investigate size and thickness variation to assess morphometric patterns underpinning the production of the participants at the beginning and the end of the training. The morphometric analysis included height and thickness. Thickness was measured at the centre of the base and along three spots of the walls corresponding to the lower, medium and higher parts of the vessel. Differences in the dimension of the experimental vessels produced before and after the training were assessed through paired Student’s t-tests.

#### Qualitative analysis of the vessels

Qualitative analysis of the experimental pottery consists of the description of the ceramic surface where traces of the potter’s gestures and tools can be observed and used as a means to infer craft behaviors [7,14]. General topography and manufacturing traces impressed along the pottery surface reflect the shaping technique [7] and the dexterity of the potter [15,60]. We used these features to correlate the ability of the participants and the technological traces they frequently leave during the shaping process. The technological traces analysis availed of description criteria developed in former studies [7,14,15,60,61] and adapted to the experimental pottery collection. Criteria selected to assess the quality of the experimental production include presence/absence of collapse of the basis, presence/absence of drying crevices, presence/absence of coils’ junctions on the surfaces, internal and external topography (flat, sinuous, uneven), presence/absence of surface treatment (no treatment, smoothing), shape, frequency and direction of technological traces (dermatoglyphics, batches of striations, depressions) (**S2 Table**).

## Results

Twenty-eight healthy individuals participated in the study (n = 13 experimental group). All participants were tested in two separate sessions, with an average gap of 44 days between sessions (range of days = 33–63, SD = 16.8). During both sessions, participants viewed video clips lasting 1557 ms depicting an expert performing coil making and coil surface treatment. In half of the video clips there was an error committed by the expert regarding the object making. During the viewing of the stimuli, EEG-TMS co-registration was carried out to monitor brain activity and motor excitability. Participants in the experimental group underwent a neolithic pottery technique training between two sessions, receiving theoretical lessons and hands-on practice in shaping coils and vessels under the guidance of an expert. Control group participants did not attend any lessons. TMS-pulse was applied on the left primary motor cortex of participants and electromyography (EMG) was recorded from the first dorsal interosseous and opponens pollicis muscles of the participants’ right hand. Moreover, all participants were asked to shape coils and vessels after both sessions and variations in these final products were qualitatively and quantitatively analysed.

### MEPs

**Fig 3:**
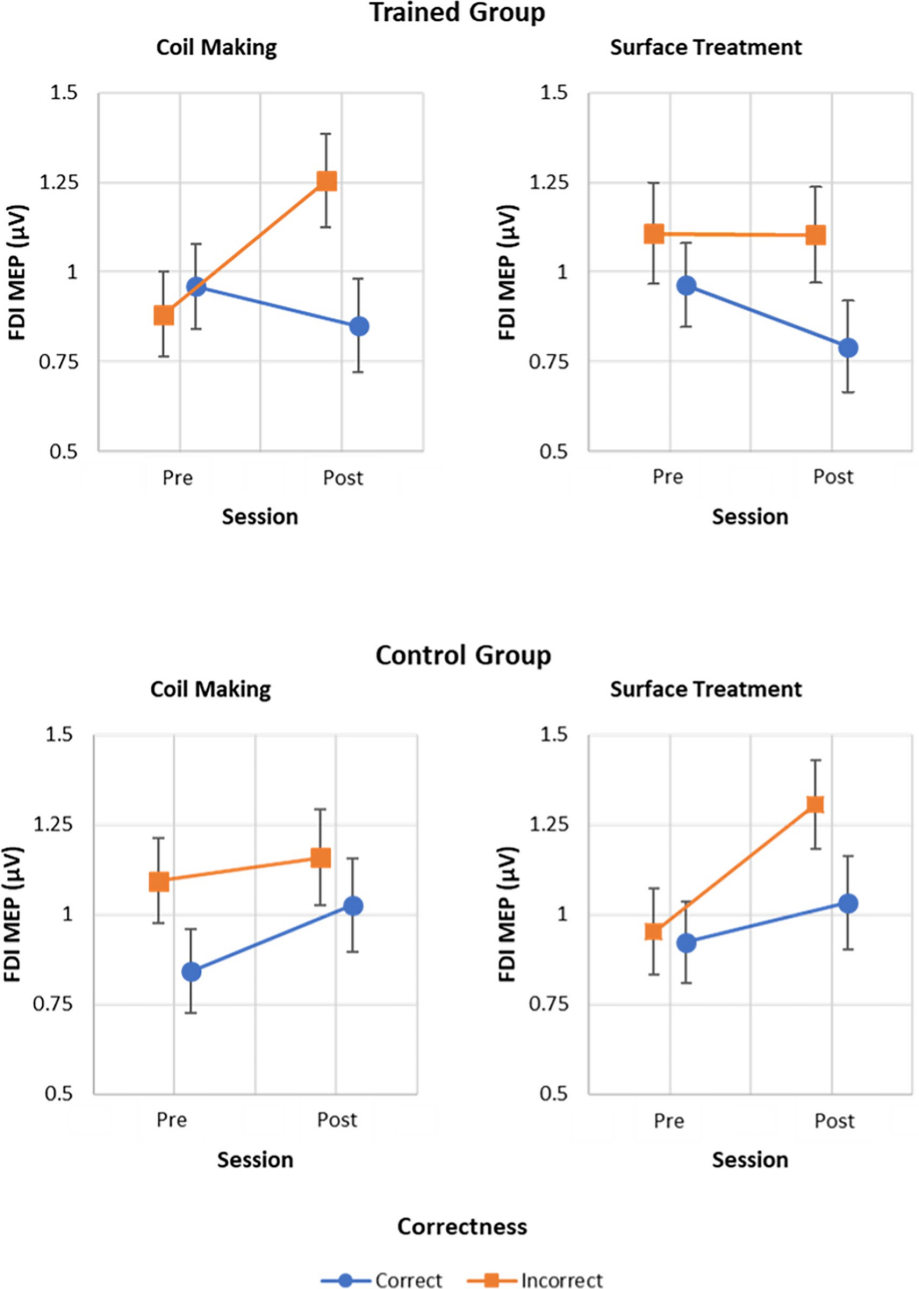
Interact plot for the LMM of first dorsal interosseus’MEPs. The figure shows the estimated marginal means of the normalised MEP amplitude for the GROUP x SESSION x TECHNIQUE x CORRECTNESS interaction. Error bars represent standard errors.

MEP for hand muscles were analysed using linear mixed-effects models (LMM) (see MATERIALS AND METHODS). A summary of the LMM results for the index finger muscle is presented in **Table 1**. Results showed a significant Group (Trained vs. Control) x Session (Pre vs. Post) x Correctness (Correct vs. Incorrect) interaction (p = .037). Although the Group x Session x Technique (Coil Making vs. Surface Treatment) x Correctness interaction was not significant (p = .100), uncorrected pairwise comparisons revealed different patterns for the two techniques (**Fig 3**). Regarding the Coil Making technique, the trained group exhibited a higher MEP amplitude in response to incorrect actions during the second session compared to the first one (t(1214) = 2.643, p = .008)), as well as a significant difference in the second session between correct and incorrect actions (t(1214) = 2.664, p = .008). These differences were not significant in the control group (ps >.406). Concerning the control condition Surface Treatment, the trained group exhibited a significant difference in the second session between correct and incorrect actions (t(1202) = 2.028, p = .043), but the between-sessions difference in MEPs following incorrect actions was not significant (t(1221) = -0.026, p = .980). The opposite pattern was observed for the control group, with a significant between-sessions difference in MEPs following incorrect actions (t(1218) = 2.440, p = .015), but a non-significant difference in the second session between correct and incorrect actions (t(1204) = 1.807, p = .071).

**Table 1:** Summary output of the LMM of first dorsal interosseus’ MEPs.

Predictors	Estimates	CI	p
(Intercept)	0.84	0.61–1.07	< .001
Correctness	0.25	-0.01–0.52	.059
Technique	0.08	-0.17–0.34	.531
Session	0.19	-0.10–0.47	.209
Group	0.12	-0.21–0.45	.471
Correctness × Technique	-0.22	-0.60–0.15	.240
Correctness [1] × Session	-0.12	-0.53–0.29	.561
Technique × Session	-0.07	-0.47–0.33	.715
Correctness × Group	-0.33	-0.70–0.03	.075
Technique × Group	-0.08	-0.44–0.28	.668
Session × Group	-0.29	-0.70–0.11	.150
Correctness × Technique × Session	0.36	-0.20–0.93	.209
Correctness × Technique × Group	0.45	-0.09–0.99	.105
Correctness × Session × Group	0.6	0.04–1.17	**.037**
Technique × Session × Group	0.01	-0.54–0.57	.962
Correctness × Technique × Session × Group	-0.68	-1.49–0.13	.100

Regarding the LMM results for the control muscle (**Table 2**), the results did not show any significant differences in MEP amplitude between sessions and groups.

**Table 2:** Summary output of the LMM of opponens pollicis’ MEPs.

Predictors	Estimates	CI	p
(Intercept)	0.63	0.48–0.79	< .001
Correctness	0.23	0.04–0.41	**.018**
Technique	0.2	0.01–0.38	**.036**
Session	0.09	-0.13–0.31	.417
Group	0.2	-0.02–0.43	.075
Correctness × Technique	-0.21	-0.48–0.05	.119
Correctness [1] × Session	-0.07	-0.38–0.25	.675
Technique × Session	-0.1	-0.41–0.20	.512
Correctness × Group	-0.08	-0.34–0.18	.545
Technique × Group	-0.18	-0.44–0.09	.189
Session × Group	-0.06	-0.36–0.23	.671
Correctness × Technique × Session	0.14	-0.29–0.58	.515
Correctness × Technique × Group	0.17	-0.22–0.56	.397
Correctness × Session × Group	-0.03	-0.45–0.39	.884
Technique × Session × Group	0.01	-0.40–0.43	.949
Correctness × Technique × Session × Group	-0.10	-0.70–0.50	.738

### EEG

Global Field Power (GFP), which is a measure of brain activity, was computed to identify the time-windows and scalp locations of interest for inferential analysis (see MATERIALS AND METHODS).

**Fig 4:**
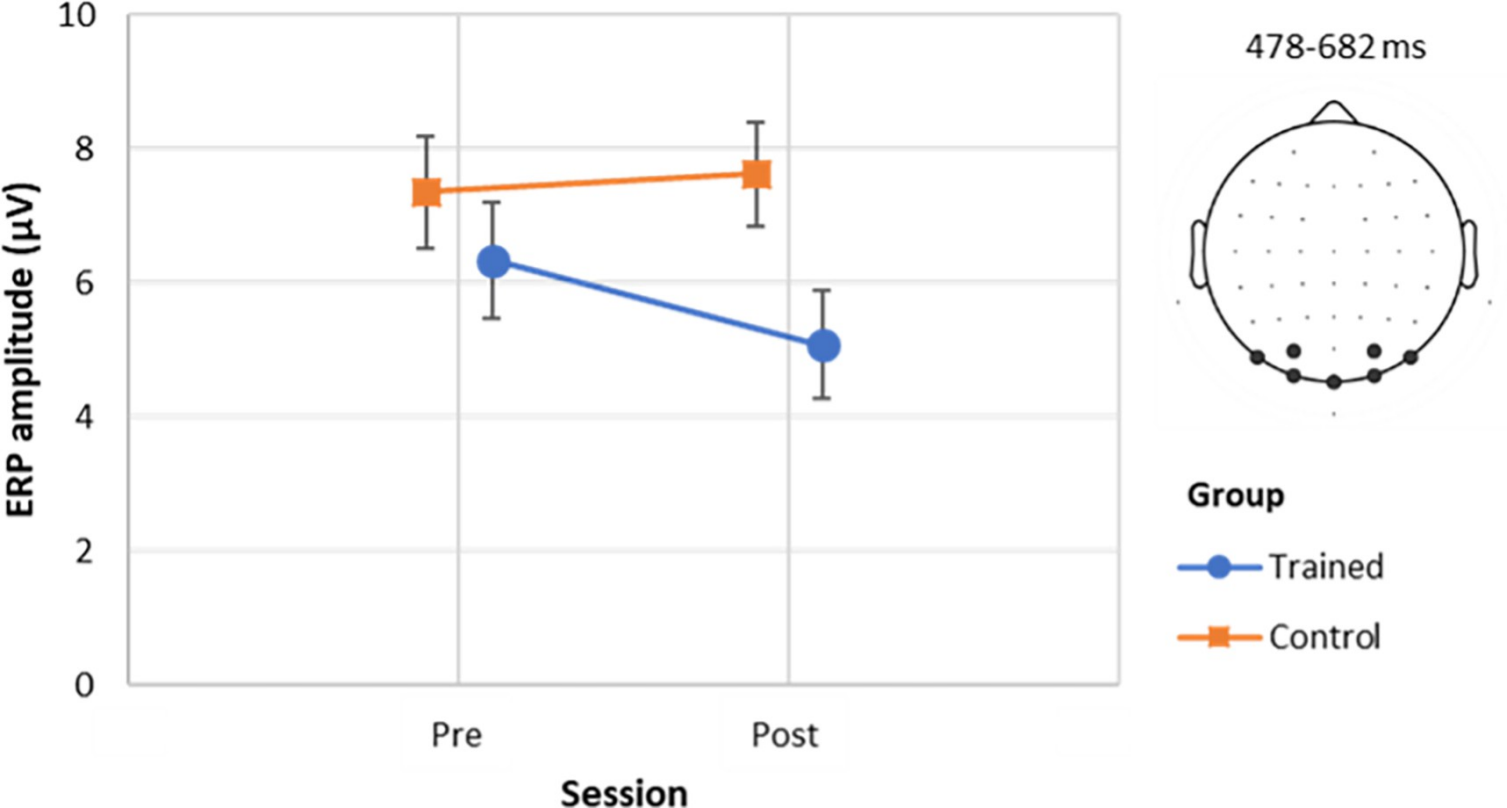
Interact plot for the EEG analysis of the first time-window of interest (action observation). The figure shows the estimated marginal means of the ERP amplitude averaged across the identified channels (PO7, PO3, PO4, PO8, O1, Oz, O2) and time-points (from 478 ms to 682 ms) of interest for the 2 groups x 2 sessions interaction. Error bars represent standard errors.

A first ERP component was identified in the time-window from 478 ms after the video onset to 682 ms over posterior electrodes (PO7, PO3, PO4, PO8, O1, Oz, O2). This ERP occurred during the observation of the actions performed by the expert. The results of the Group x Session mixed ANOVA did not reveal any significant effects (**Table 3**). However, uncorrected post-hoc pairwise comparisons did reveal a between-group difference during session 2 (t(25) = -2.258, *p* = .033). As shown in **Fig 4**, the trained group seemed to exhibit a lower ERP amplitude during the second session compared to the control group.

**Table 3:** Results of the 2 groups x 2 sessions mixed ANOVA of the first time window of interest (action observation).

		Sum of Squares		df		Mean Square		F		p	
***Within Subjects Effect***											
SESSION		3.36		1		3.36		0.854		0.364	
SESSION✻ GROUP		7.86		1		7.86		1.994		0.170	
Residual		98.50		25		3.94					
***Between Subjects Effect***											
GROUP		42.8		1		42.8		**2.93**		**0.099**	
Residual		364.5		25		14.6					

*Note*. Type 3 Sums of Squares.

A second ERP was observed from 74 ms to 230 ms after TMS pulse deliver over a frontocentral (Fz, F2, F4, FC1, FC2, FC4, Cz, C2) and a left-lateral (FT7, T7, C5, TP7, CP5, CP3, P7, P5) cluster. This time-window occurred after action completion, with an error in half of the trials. The results of the Groups x Sessions x Correctness mixed ANOVA did not reveal any significant effects for both clusters (Tables 4 and 5, respectively). Uncorrected post-hoc pairwise comparisons did not reveal any significant between-group differences (all p>.067).

**Table 4:** Results of the 2 groups x 2 sessions x 2 correctness mixed ANOVA of the second time window of interest (TMS pulse deliver) in the first cluster (Fz, F2, F4, FC1, FC2, FC4, Cz, C2).

		Sum of Squares		df		Mean Square		F		p	
***Within Subjects Effects***											
Session		0.2439		1		0.2439		0.0194		0.890	
Session ✻ GROUP		0.3138		1		0.3138		0.0250		0.876	
Residual		314.2974		25		12.5719					
Error		0.6428		1		0.6428		0.5274		0.474	
Error ✻ GROUP		0.1732		1		0.1732		0.1421		0.709	
Residual		30.4712		25		1.2188					
Session ✻ Error		1.9134		1		1.9134		4.1455		0.052	
Session ✻ Error ✻ GROUP		0.0923		1		0.0923		0.1999		0.659	
Residual		11.5391		25		0.4616					
***Between Subjects Effects***											
GROUP		10.0		1		10.0		0.627		0.436	
Residual		399.5		25		16.0					

Note. Type 3 Sums of Squares.

**Table 5:** Results of the 2 groups x 2 sessions x 2 correctness mixed ANOVA of the second time window (TMS pulse deliver) of interest in the second cluster (FT7, T7, C5, TP7, CP5, CP3, P7, P5).

		Sum of Squares		df		Mean Square		F		p	
***Within Subjects Effects***											
Session		22.3835		1		22.3835		1.9071		0.180	
Session ✻ GROUP		1.5843		1		1.5843		0.1350		0.716	
Residual		293.4258		25		11.7370					
Error		0.4554		1		0.4554		0.9361		0.343	
Error ✻ GROUP		0.2666		1		0.2666		0.5479		0.466	
Residual		12.1627		25		0.4865					
Session ✻ Error		0.0898		1		0.0898		0.1796		0.675	
Session ✻ Error ✻ GROUP		0.0343		1		0.0343		0.0686		0.796	
Residual		12.5039		25		0.5002					
***Between Subjects Effects***											
GROUP		21.4		1		21.4		1.30		0.265	
Residual		410.1		25		16.4					

Note. Type 3 Sums of Squares.

### Qualitative analysis of coils

**Fig 5:**
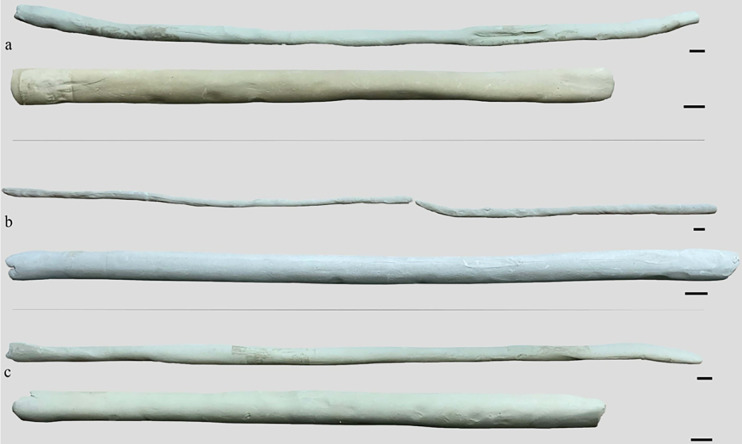
Comparison between coils made before the training and at the end of the training. a-c: Coils produced by three different participants (black bar is 1 cm).

Coils shaped by both the groups before the training are highly irregular in shape and thickness. Sub-circular or flattened cross-sections are caused by inexperience in managing the combination of rolling gestures and pressure to shape the lump of clay. Consistent variations of thickness, resulting in highly wavy profiles (**Figs** 5 **and S1**), recur along the whole profile and at the extremities of the coil due to the heavy pressure of the fingers.

After the training changes in qualitative features were observed in the trained group. Coils were more homogeneous in shape and thickness along the whole coil and in particular the extremities, which appear rounded (**Figs** 5 **and S2**).

### Geometric morphometric analysis of coils

The morphological differences between the shape of the coils produced before and after the training session, which were identified qualitatively, are confirmed also through GMM analysis. When analysing the shapes of the coils produced by the subjects of the training group 21 harmonics were selected.

**Fig 6:**
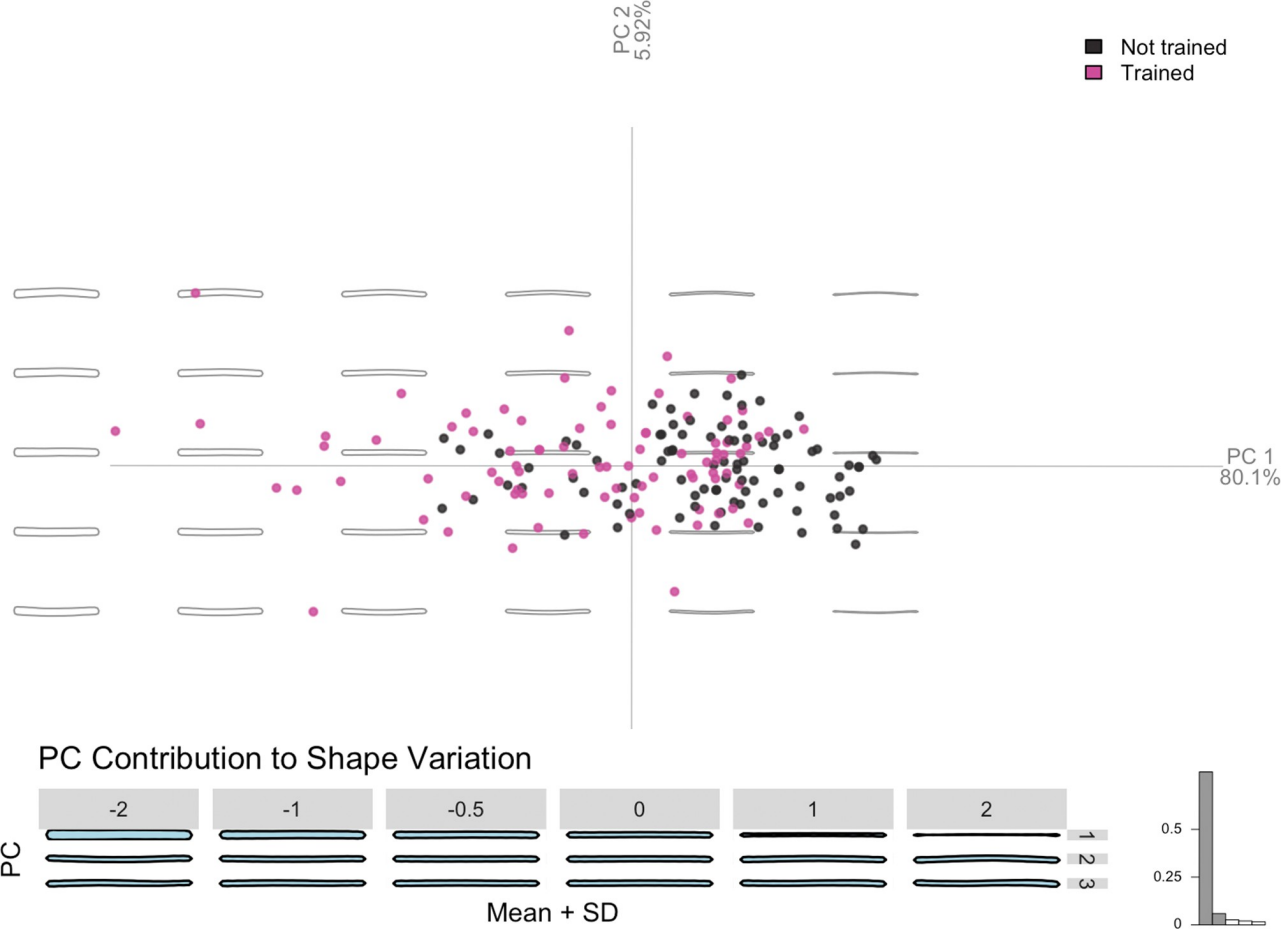
Significant differences in the shape of the coils. Significant differences were identified through MANOVA, performed both on the elliptic Fourier coefficients (p = <0.001) and the PCA scores (p = <0.001). More specifically, significant differences between the coils produced before and after the training were shown performing a MANOVA Pairwise, which returned a p value of <0.001 on the PCA scores.

As shown in **Fig 6** the first two components of the PCA performed on the elliptic Fourier coefficient are able to explain 82% of the variation in shapes observed in the coil assemblage.

Such variation relies mostly on the thickness and regularity of the coils which is explained mostly through Principal Component 1. Indeed, most of the coils produced before the training result to be thin and irregular, while the ones produced after the training are thicker and more regular (**Fig 6**). A further aspect of differentiation is given by the straightness of the coils, which refers to Principal Component 2 and accounts for the 5.92% of shape variation. More curved coils are produced before the training, while straighter ones are commonly manufactured after the training sessions (**Fig 6**).

Significant differences in the shape of the coils were identified through MANOVA, performed both on the elliptic Fourier coefficients (p = <0.001) and the PCA scores (p = <0.001). More specifically, significant differences between the coils produced before and after the training were shown performing a MANOVA Pairwise, which returned a p value of <0.001 on the PCA scores. Then the same analyses were performed on the shape of the coils produced by subjects of the control group. In this case, the first two Principal Component allow to define only the 57.8% of variation characterising the assemblage (**Fig 6**). Through MANOVA, no significant differences emerge within the coil assemblage (p = 0.8715) and between the two phases of production (p = 0.1276).

The increase in the thickness and homogeneity of coils and vessels’ walls suggests the newly acquired capacity of the subjects to manage the pressure while rolling the lump of clay and pinching the vessel’s walls. The trained participants learned to measure the right pressure enough to shape the paste without deforming it. The experimental framework highlighted clear differences between subjects in making both coils and pots.

### Quantitative features of the vessels

We found a variation in the mean height of the vessels produced by the participants at the beginning and the end of the training. The mean height of the vessels at the beginning of the training was 91.2 mm. The mean height of the vessels at the end of the training corresponded to 121 mm. A significant difference was recorded comparing the height of the vessels produced before and after the training (p = 0.008842).

Regarding the mean thickness of the vessel, variations were found only in specific spots of the walls. The mean thickness of the base at the beginning of the training was 10.8 mm, while the mean thickness at the end of the training increased, reaching 13.0 mm.

A variation was found in the mean thickness of the vessel walls. Changes were observed at the lower, medium, and higher parts. The lower part of the walls was 9.65 mm at the beginning of the training and 12.8 mm at the end of the training. The medium part of the walls corresponded to 8.41 mm at the beginning of the training mm and 10.7 mm at the end of the training. The higher part of the walls, which coincides with the rim, was 7.23 mm at the beginning of the training and 8.96 mm at the end of the training.

Comparing the values of the objects produced before and after the training, a significant difference was recorded for the lower (p = 0.01797) and medium (p = 0.007504) thickness of the vessels. No significant difference was found in the thickness of the base (p = 0.09866) and the rim (p = 0.08572). We can observe an increase in the height of the vessels and a thickening of walls at the end of the training. The improvement specifically consisted of the increase in the mean height and thickness of the vessels produced at the end of the training. This confirms the embodiment of a new set of gestures used with efficacy to shape functional objects.

### Qualitative features of the vessels

**Fig 7:**
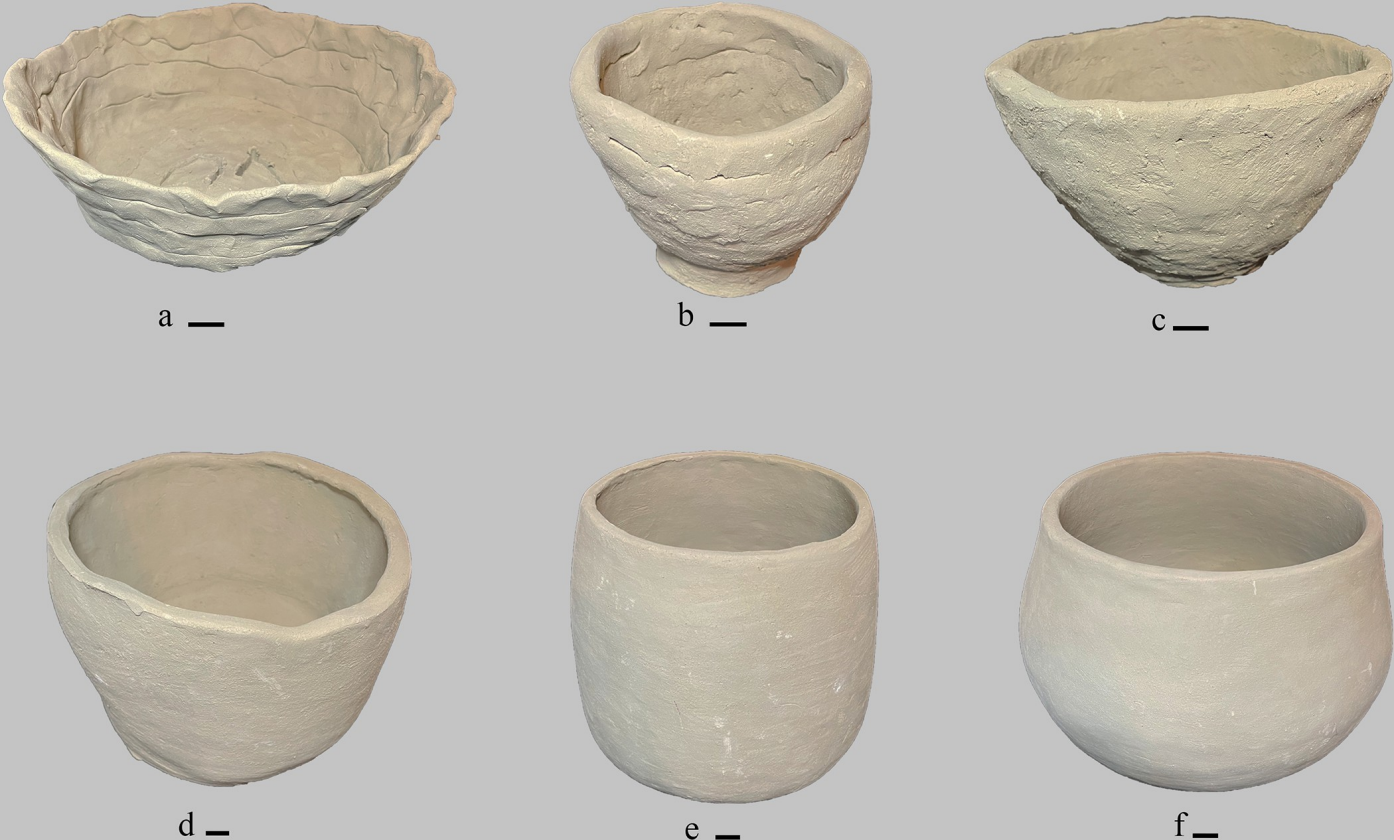
Comparison between experimental vessels produced at the beginning and at the end of the training. a-c: Vessels shaped at the beginning of the training; d-f: Vessels shaped at the end of the training. The vessels made by three diverse participants were selected to show the improvements achieved at the end of the training (black bar is 1 cm).

The most common mistakes observed in the production of the participants at the beginning of the training consisted of 1. collapsing of the basis, 2. superficial crevices and 3. incomplete shaping (**Figs 7A–7C** and **S3**) (**S2 Table**).

We observed the basis collapse on half of the vessels (n = 6). It consisted of the isolated or diffused lowering of the bottom. The pressure of the higher part of the vessel, combined with a high moisture level of the clay paste, were the leading causes of the basis collapse. This mistake only in three cases was replicated by the same participants. The development of crevices was another recurring mistake, even with a low incidence (n = 3), in the shaping process (**S3B Fig**). This surface anomaly consists of isolated or multiple superficial crevices, often localised along the rim and caused by a sudden surface drying during the shaping process. The drying of the clay paste was a consequence of the heat of the hands pinching and shaping the same area of the container until it dried. Nevertheless, this mistake was considerably reduced in the final products. The third mistake is the incompleteness of the shaping sequence that generally interrupts the building phase, which coincides with the overlapping of coils. Most of the vessels made by the participants at the beginning of the training (n = 11) show junctions among coils, consisting of horizontal depressions formed at the superimposition of a coil to another (**Figs 7A–7C** and **S3A**). Novices superficially joined these spots, leaving the junctions partially or completely visible. Surface finishing as smoothing was also absent in all the vessels (n = 13) (**S3B and S3C Fig**).

This combination caused irregular surface topographies (**Fig 7A–7C**), characterised by elongated or pinched dermatoglyphic traces, reflecting the incomplete manufacturing sequence and making the vessel weak and exposed to mechanical stress. These mistakes were considerably reduced at the end of the training when the participants (N = 10) embodied the coils junction step and completed the shaping process, smoothing the surface (N = 13).

The basis collapse is a frequent mistake that is difficult to amend among naïve and beginners (**S3D–S3E Fig**). Managing the constraints of the clay moisture may require a longer experience. The low capacity to manage the clay paste can also be considered the cause of crevice formation. The low efficacy of the novices’ shaping gestures leads them to over-shape the same spot of the vessel, as the rim, until they unintentionally get it dry under the warmth of their hands. Except for the collapse of the basis, which appears to be a mistake requiring time to be overcome, we recorded a consistent reduction of creviced and other surface mistakes (e.g., visible coil junctions or uneven surfaces) in the pottery production at the end of the training. Although all the participants embodied the correct manufacturing sequence, from coil shaping to surface treatment of the final product refined using tools, differences in dexterity could be observed in the irregularity of the surface topography, and it varied among individuals (**Figs 7D–7F** and **S4**). Most of the participants shaped vessels with sinuous external surfaces (N = 7), while a few individuals made vessels with uneven (N = 2) and flat external surfaces (N = 4) (**S4 Fig**). This latter group coincides with the individuals that most developed dexterity in managing the constraints of surface plasticity and irregularity obtaining well-refined products.

The trained participants learned to manage the constraints of the paste plasticity and reduce or avoid the risk of collapse which is a frequent mistake in novices. The newly acquired ability to shape clay into homogeneous and right-size coils favours the production of vessels with a more solid structure and of a higher aesthetical value.

## Discussion

Mechanisms such as perceptual-motor and cognitive control and working memory were essentials during the early phases of human evolution [26,62–65] and likely played a key role also during the gradual adoption and diffusion of ceramic technology. Based on the Material Engagement Theory [66,67], clay is not a passive raw material shaped by the potter at his/her necessity; it is an active part of a mechanism of co-construction [29] in which the effect between potter and clay is ideally mutual. From this material engagement perspective, our interdisciplinary research addresses, for the first time, the neural plasticity of the primary motor cortex of early potters. With this purpose, we monitored the neural and behavioural changes of two groups, one composed of *naïve* participants trained intensively in coiling, among the earliest neolithic pottery techniques, and a control group not involved in pottery training. We assessed the impact of the newly acquired skill monitoring the participants while watching videos of mistakes as well as correct gestures involved in coil manufacturing. Trained and untrained participants underwent sessions of TMS-EEG co-registration scheduled at the beginning and at the end of the training. Along with the TMS-EEG recording, we compared quantitative and qualitative morphological features of the handcrafts produced by the two groups (coils made by both groups during the experiment and coiled vessels shaped only by the participant during the intensive training) to assess, on one hand, the neural changes and, on the other hand, which features on the experimental replicas associated to the improvements in pottery making can then be useful to interpret the archaeological assemblages in terms of potters’ skill.

Although this is a preliminary contribution, the study shows the potential of integrating archaeology and cognitive neuroscience to investigate the reciprocal effect of ancient technologies and craftspeople, provide new insights into understanding variations in material culture, and infer past behaviors in terms of motor skills and social learning.

Differences in neural changes were found between the two groups of participants involved in the study. In particular, a higher corticospinal excitability was recorded in response to incorrect actions in the second session of the trained group compared to the first one and a significant difference was exhibited in the second session between correct and incorrect actions. These differences were not significant in the control group. Upon examining GFP, we identified an ERP that occurred 478 ms after the onset of the video and lasted for 204 ms. This time-window corresponds to the observation of actions, and any distinct activation between the groups within this timeframe indicates different processing of the observed actions. Participants in the experimental group, who underwent training on pottery making, exhibited a different activity pattern characterized by a lower amplitude in the ERP during the action observation time-window. This difference only showed up in the second session, suggesting that participants in the experimental group processed motor skills related to pottery making differently from the control group who lacked practical training. This result implies that motor skill training employed in the present study might have modulated the underlying cognitive processes related to the observation of motor actions. Overall, these neural findings are in line with previous studies demonstrating that action observation modulates brain activity depending on the motor experience of the observer [37,68–71]. They thus represent a promising starting point to investigate, under a neuroarcheological framework, how motor training and acquired motor skills can alter the structural and functional properties of the brain [72,73]. A final note concerns the fact that the detected between-group differences were observed at the occipital scalp sites. This might reflect differences in the coding and transmission of information from the visual to the motor region as the direct-matching hypothesis [74] suggests a reciprocal relationship between the visual and motor systems in action understanding.

Concerning ERP responses associated with error monitoring, we did not observe any significant modulation or between-group and between-session differences. Considering that error-related effects were observed in the MEP signals, this lack of differentiation can be plausibly attributed to the limited number of error-displaying trials, which poses a threat to ERP studies and Error-related Negativity [75–79]. Unfortunately, the lack of significant findings in the second analysis window following TMS pulse delivery limited the impact of the pilot study on the co-registration aspects.

Based on the preliminary results of the neural and behavioural trials we found that the intensive practice in the coiling technique, requiring a specific hand movement and the capacity to manage the right finger pressure to shape the clay paste without deforming it, led to a change in the functional properties (i.e., corticospinal excitability) of brain areas responsible for action control and execution, including the end-point of the cortical motor system, namely the primary motor cortex. The change was recorded in participants trained in coiling twice to three times a week in a month for an average of 27 hours.

This suggests that a four-week training period is sufficient for beginners trained by an expert to gain basic proficiency in modeling coiled vessels. This time frame also finds correspondence with the literature regarding the minimum time required to change neuroplasticity following a practical activity [34].

Differences between the practitioners in pottery manufacture and the control group emerged also in the quantitative and qualitative features of the handcraft as coils and vessels produced at the beginning and the end of the training period. As a consequence of the mutual effect among the makers and their material productions, the more the new motor skill was acquired and embodied, the more the mistakes in their handcrafts were reduced. The quantitative and qualitative analysis of the experimental handcrafts showed an improvement in the participants’ skills observed in the presence/absence of structural and aesthetical mistakes in coils and vessels shaped at the beginning and at the end of the training. Changes were observed in the shape of the coils and specifically in their thickness and regularity. Before the training, in both groups, the coils were thin, irregular and curved. After the training, the coils were thicker, homogeneous and straight in shape. Conversely, the coils of the control group remained the same in both sessions showing high irregularity in thickness and shape.

Most of the participants in the training group kept reducing recurring novice mistakes such as the deformation (a mistake that causes later the irregularity of the vessel wall) and breakage of the coils during rolling, the collapse of the basis and the incompleteness of the shaping sequence corresponding to the coil’s junctions visible along the vessel’s walls.

The transmission of technical knowledge and suggestions on how to improve gestures and correct mistakes reduced the time necessary to explore the potential of the material and manage the constraints (e.g. plasticity) to achieve the basics of this technology. Conversely, mastering the technology, such as the ability to make more complex shapes and highly refined vessels, requires time and it is favoured by former experience in activities involving similar gestures and/or providing feedback for developing the new skill [22].

In a general framework, the neural and behavioural tests allowed us to confirm that pottery manufacturing is a skill that requires time to be acquired and mastered. It is not a straightforward skill and its acquisition and mastering are also a consequence of the social learning systems and cultural contexts [80–83].

The training conditions adopted during our experiment consisted of group work during which the novices imitated and interacted with each other and with the expert. This promoted learning of the technical sequence by reducing errors and improving the quality of the final product. By observing the increase in the quality and quantity of pottery during the Neolithic, we can speculate that production was modulated by a mode of knowledge transfer compatible with that tested by our research, highlighting the role that experts may have played in the transmission of knowledge in prehistoric communities. Based on these hypotheses and scientific evidence, future research will explore the possible relationship between increased production quality, producers’ dexterity, the learning system and sedentism. Based on a hypothetical bio-cultural feedback loop [22], the existence of prior skills might have promoted the development and consolidation of new skills at different times among individuals. We hypothesize that the acquisition of the correct gestures for evenly rolling a lump of clay might have influenced the dexterity of early potters by refining the motor cortical representation of trained hand muscles. Later, the continued adoption of this new technology may have permanently altered the functional characteristics of the motor cortex of skilled potters, also influencing the cultural, social and economic dynamics underlying the neolithic technological transition and the craft specialisation.

The transmission, through mechanisms of cumulative culture [84], of motor skills necessary to efficiently shape clay among neolithic communities, laid the foundations for developing diverse abilities required by following technological innovations, such as the potter’s wheel. Clay shaping skills, along with similar activities, could have played a key role in the bio‐cultural feedback loops [22] that supported the acquisition of new craft abilities.

This research provides also essential insights regarding archaeological interpretation. The assessment of the skill in trained subjects and the characterization of features of naïve skill suggests that the qualitative and quantitative features of the vessels reflect not only the cultural traditions of a community [7] in terms of the way of doing and shaping techniques, but they are also the direct consequence of the potter’s skill [24,85]. The results of the present research show that the experience and motor skills of potters modulate the variability of material culture. Moreover, the errors do not necessarily indicate the product of children but could be the product of potters without training or lacking previous experience that could support further improvements in pottery production. Our results provide promising insights regarding the effect of new motor skill acquisition on neuroplasticity and how these plastic changes can be revealed with an action observation task and neural techniques. The small sample size precluded us from exploring potential correlations between measures evaluating the effectiveness of the training and the neural correlates obtained through TMS-EEG co-registration. Future comparisons with higher sample size will also explore the association between outperformer and neuroplasticity. By expanding the sample size and the number of trials, a comparison between pottery modeling and other crafts, such as flintknapping or basketry, could also be proposed in the future, allowing us to assess whether the same results are observed consistently or whether variations emerge between crafts. This comparison could be the key to understanding technological transitions over the past and how these impacted human behaviours, especially regarding knowledge transmission, learning mechanisms and craft specialisation. Moreover, our paradigm is also potentially very useful for future studies investigating technique acquisition in different age groups [83], and, widely, to develop reliable and shared criteria to assess craft skills in material culture. Therefore, considering the results of qualitative and quantitative analysis of experimental artefacts at the beginning and end of specific training, the research is promising for future studies on the co-evolution of human abilities and technology.

## Conclusions

This interdisciplinary study combined experimental archaeology, cognitive neuroscience, and behavioural techniques to study the neural plasticity of the primary motor cortex resulting from the development of new skills in pottery making, as probably occurred in neolithic potters.

Through a technique of TMS-EEG co-registration, we examined neural indices before and after training, finding differences among subjects trained in coiling compared to the control group. Intensive coiling training, used as a proxy of the foremost potter’s activity during the Neolithic, modified the functional properties of a brain area responsible for action control and execution. In behavioural terms, participants exhibited improved motor skills and reduced mistakes in their artefacts. These preliminary results suggest that the acquisition and mastery of ceramic skills were probably influenced by social learning systems and cultural contexts. Moreover, the changes in the participants’ artefacts suggest that motor skills consistently impact material culture’s variability. Therefore, despite the preliminary nature of the research, the findings offer promising insights into the effects of motor skill acquisition on neuroplasticity and suggest avenues for future studies on the co-evolution of human abilities and technology.

## Supporting information

S1 Fig Details of coils made by participants before the training. Coils are irregular in shape and thickness (black bar is 1 cm).
(PNG)

S2 Fig Details of coils made by participants after the training. Coils are regular in shape and thickness (black bar is 1 cm).
(PNG)

S3 Fig Characteristics and distribution of errors made by participants along the vessels. a. visible horizontal coils’ junctions along internal and external walls (incomplete shaping); b: crevices above the external rim; c: uneven external surface as a result of lacking surface treatment (incomplete shaping); d-e: collapse of the vessel base during the vessel’s shaping (black bar is 1 cm).
(JPG)

S4 Fig a-e: features and distribution of technological traces and reduction of mistakes of the participants at the end of the training.
(JPG)

S1 Table Experience and activity performed by the participants: Quantification of the experience (annual frequency, 0–12) in activities requiring gestures similar to the coiling technique, quantification of the training in coiling technique, amount of coiled shaped during the training, total of vessels shaped using the coiling technique at the end of the training period. The two groups did not show any significant difference (p = 0.258) in their former experience.
(DOCX)

S2 Table Qualitative features of the vessels shaped by the participants at the beginning of the training and the end of the training. (DOCX)
