# Supplementary figures and images for: Neural plasticity in early potters: Shape analysis and TMS-EEG co-registration trace the rise of a new motor skill
Source: PLoS One. 2025 Jan 17;20(1):e0316545. doi: 10.1371/journal.pone.0316545 (PMC11741608; doi:10.1371/journal.pone.0316545)

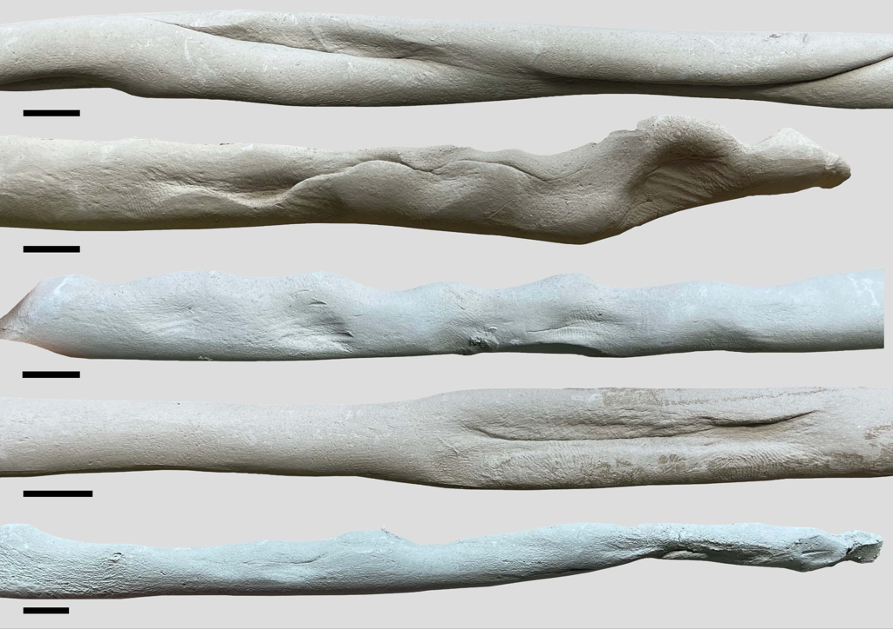

Supplement: S1 Fig — Coils are irregular in shape and thickness (black bar is 1 cm). (PNG) [file pone.0316545.s001.png]

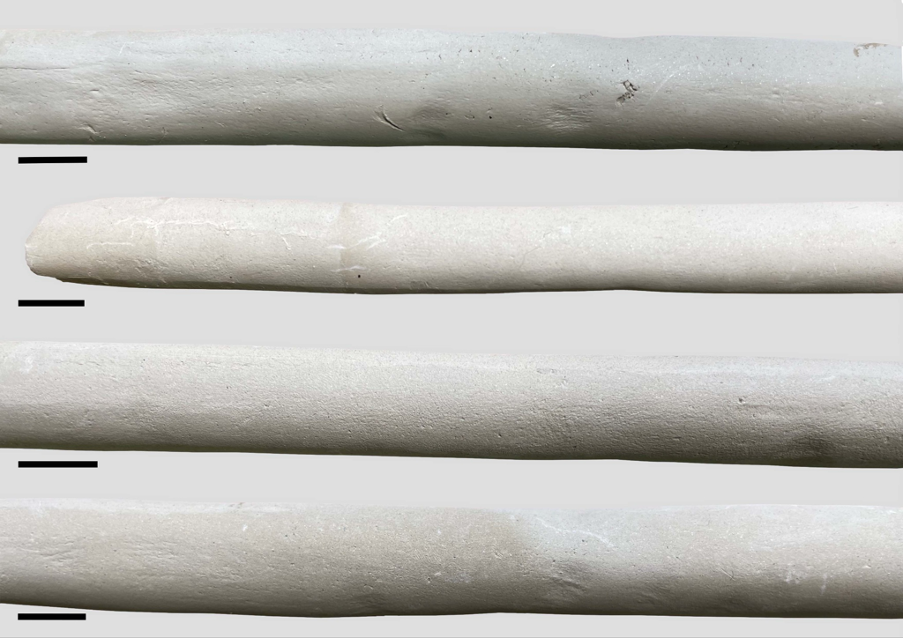

Supplement: S2 Fig — Coils are regular in shape and thickness (black bar is 1 cm). (PNG) [file pone.0316545.s002.png]

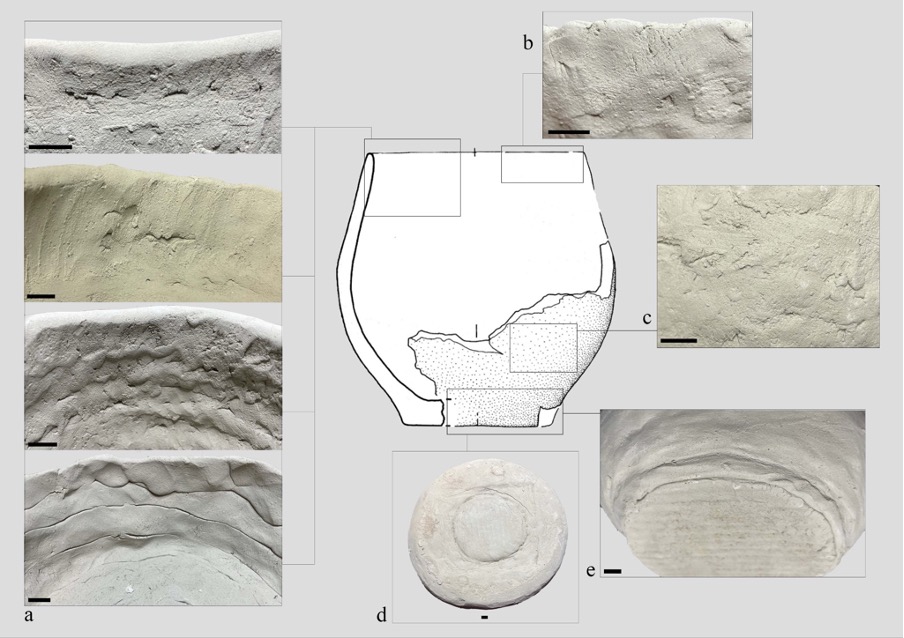

Supplement: S3 Fig — a. visible horizontal coils’ junctions along internal and external walls (incomplete shaping); b: crevices above the external rim; c: uneven external surface as a result of lacking surface treatment (incomplete shaping); d-e: collapse of the vessel base during the vessel’s shaping (black bar is 1 cm). (JPG) [file pone.0316545.s003.jpg]

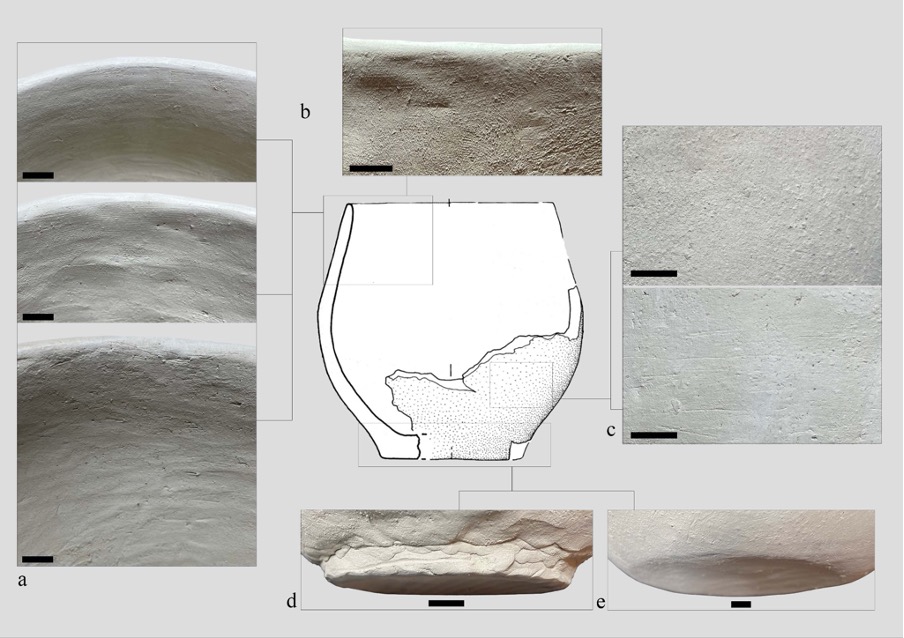

Supplement: S4 Fig — a-e: features and distribution of technological traces and reduction of mistakes of the participants at the end of the training. (JPG) [file pone.0316545.s004.jpg]

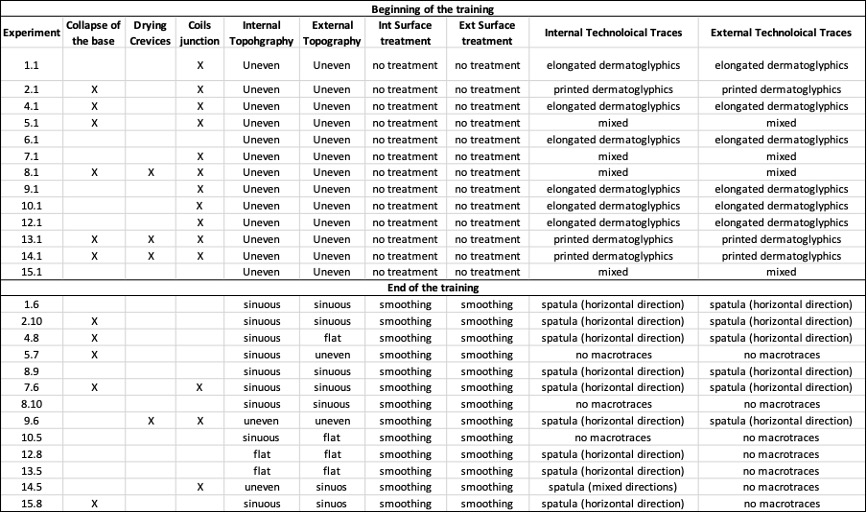

Supplement: S2 Table — (DOCX) [file pone.0316545.s006.docx]
