# Supplementary material for: Neural plasticity in early potters: Shape analysis and TMS-EEG co-registration trace the rise of a new motor skill
Source: PLoS One. 2025 Jan 17;20(1):e0316545. doi: 10.1371/journal.pone.0316545 (PMC11741608; doi:10.1371/journal.pone.0316545)
Supplement: S1 Table — The two groups did not show any significant difference (p = 0.258) in their former experience. (DOCX) [file pone.0316545.s005.docx]

| **Subject** | **coiling gesture former experience** | **Pottery training (h)** | **Coils shaped** | **Vessels produced** |
| --- | --- | --- | --- | --- |
| 2 | twice a year | 28.30 | 90 | 10 |
| 3 | twice a year | 42 | **114** | 10 |
| 4 | once a month | 31.30 | 67 | 10 |
| 5 | twice a year | 30 | 89 | 8 |
| 7 | once a year | 27 | 72 | 8 |
| 8 | twice a year | 19 | 77 | 9 |
| 9 | once a year | 28.30 | 75 | 8 |
| 10 | once a year | 29 | 67 | 8 |
| 11 | no | 30 | 98 | 9 |
| 12 | once a year | 32.30 | **120** | 14 |
| 13 | no | 17 | 57 | 10 |
| 14 | twice a year | 22 | **122** | 11 |
| 15 | no | 32.30 | 60 | 7 |
| 1 | twice a year | 0 | 0 | 0 |
| 6 | once a year | 0 | 0 | 0 |
| 16 | twice a year | 0 | 0 | 0 |
| 17 | once a year | 0 | 0 | 0 |
| 18 | once a year | 0 | 0 | 0 |
| 23 | once a year | 0 | 0 | 0 |
| 24 | twice a year | 0 | 0 | 0 |
| 25 | 0 | 0 | 0 | 0 |
| 26 | twice a year | 0 | 0 | 0 |
| 27 | 0 | 0 | 0 | 0 |
| 28 | twice a year | 0 | 0 | 0 |
| 29 | once a year | 0 | 0 | 0 |
